# Over the rainbow: structural characterization of the chromoproteins gfasPurple, amilCP, spisPink and eforRed

**DOI:** 10.1107/S2059798322002625

**Published:** 2022-04-08

**Authors:** F. Hafna Ahmed, Alessandro T. Caputo, Nigel G. French, Thomas S. Peat, Jason Whitfield, Andrew C. Warden, Janet Newman, Colin Scott

**Affiliations:** a Land and Water, CSIRO, Clunies Ross Street, Canberra, ACT 2601, Australia; b Synthetic Biology Future Science Platform, CSIRO, Canberra, ACT 2601, Australia; c Manufacturing, CSIRO, Research Way, Clayton, VIC 3168, Australia; d The University of Queensland, St Lucia, Brisbane, QLD 4072, Australia

**Keywords:** chromoproteins, fluorescent proteins, chromophores, protein dimers, synthetic biology, GfasPurple, amilCP, spisPink, eforRed

## Abstract

Anthozoan chromoproteins are highly pigmented, diversely coloured and readily produced in recombinant expression systems. While they are a versatile and powerful building block in synthetic biology for applications such as biosensor development, they are not widely used in comparison to the related fluorescent proteins, partly due to a lack of structural characterization to aid protein engineering. Here, high-resolution X-ray crystal structures of four open-source chromoproteins, gfasPurple, amilCP, spisPink and eforRed, are presented. These proteins are dimers in solution, and mutation at the conserved dimer interface leads to loss of visible colour development in gfasPurple. The chromophores are *trans* and noncoplanar in gfasPurple, amilCP and spisPink, while that in eforRed is *cis* and noncoplanar, and also emits fluorescence. Like other characterized chromoproteins, gfasPurple, amilCP and eforRed contain an *sp* ^2^-hybridized *N*-acylimine in the peptide bond preceding the chromophore, while spisPink is unusual and demonstrates a true *sp* ^3^-hybridized *trans*-peptide bond at this position. It was found that point mutations at the chromophore-binding site in gfasPurple that substitute similar amino acids to those in amilCP and spisPink generate similar colours. These features and observations have implications for the utility of these chromoproteins in protein engineering and synthetic biology applications.

## Abstract 2

The structures of four coral chromoproteins reveal conserved dimer interfaces and insights into chromophore interactions that are important for colour development.

## 1. Introduction

Naturally occurring anthozoan (coral and sea anemone) chromoproteins and fluorescent proteins are highly pigmented and are found in a vast array of colours that are well suited to various synthetic biology applications (Alieva *et al.*, 2008 ▸). They are homologous to the well characterized *Aequorea victoria* (jellyfish) green fluorescent protein (GFP) and were first isolated from acroporid coral in 1995 (Dove *et al.*, 1995 ▸). Since then, they have been shown to be the basis of host-based pigmentation in corals and hence are the principal source of the brilliant colours observed in coral reefs (Dove *et al.*, 2001 ▸). Various anthozoan fluorescent proteins have been characterized and optimized for *in vivo* and *in vitro* imaging, including Azami-Green from *Galaxea fascicularis* (Karasawa *et al.*, 2003 ▸) and derivatives of dsRed from *Dicosoma* sp. (for example mCherry and mOrange; Shaner *et al.*, 2004 ▸).

The nonfluorescent or low-fluorescence chromoproteins in the anthozoan chromoprotein family have vivid visible pigmentation due to their high molar extinction coefficients in the visible spectrum and offer certain advantages over their fluorescent homologues in some imaging applications (Shkrob *et al.*, 2008 ▸). Like the fluorescent proteins, chromoproteins are readily produced in *Escherichia coli* and can be used to quantitatively report on gene expression (Liljeruhm *et al.*, 2018 ▸), but they can also easily be measured without the equipment required to detect fluorescence. Chromoproteins are less rapidly photobleached as they are measured at longer, less damaging wavelengths than their fluorescent counterparts (Shkrob *et al.*, 2008 ▸). Additionally, there are generally fewer cellular components that interfere with measurements of chromoproteins than of fluorescent proteins. They can be particularly effective for use as *in vivo* reporters for live cell biosensors (Liljeruhm *et al.*, 2018 ▸), as selection markers for synthetic biology (Shih *et al.*, 2015 ▸; Näsvall *et al.*, 2017 ▸) and as Förster resonance energy transfer (FRET) acceptors for photoacoustic and fluorescence lifetime imaging (Pettikiriarachchi *et al.*, 2012 ▸; Murakoshi *et al.*, 2019 ▸; Li *et al.*, 2016 ▸).

Like GFP, chromoprotein monomers have a β-barrel structure that contains a chromophore, which is autocatalytically constructed from adjacent *X*–tyrosine–glycine residues encoded within the protein sequence itself (Olenych *et al.*, 2006 ▸; Miyawaki *et al.*, 2003 ▸). In addition, they have been reported to exist as either dimers or tetramers like GFP, with an ionic protein interface on one side and a hydrophobic interface on the other (Chang *et al.*, 2019 ▸; Wall *et al.*, 2000 ▸). After protein folding, the three chromophore-forming residues cyclize to form an imidazolinone ring system that is then oxidized by molecular oxygen in the rate-limiting step for full chromophore maturation and colour development (Reid & Flynn, 1997 ▸). Colour maturation time is highly variable among different chromoprotein variants and depends on the protein folding efficiency, the accessibility of molecular oxygen and the chemical environment near the chromophore (Miyawaki *et al.*, 2003 ▸; Iizuka *et al.*, 2011 ▸). The latter also influences the colour of the protein, along with the exact amino-acid composition of the chromophore as well as its *cis*/*trans* conformation and coplanarity, which determine the extent of π-orbital conjugation and absorption wavelength (Olenych *et al.*, 2006 ▸). In chromoproteins, the chromophore has so far been observed to adopt *trans* noncoplanar conformations (Chan *et al.*, 2006 ▸; Wannier & Mayo, 2014 ▸; Chang *et al.*, 2019 ▸; Chiang *et al.*, 2015 ▸; Gurskaya *et al.*, 2001 ▸; Andresen *et al.*, 2005 ▸; Henderson & Remington, 2006 ▸), whereas they are usually *cis* coplanar in fluorescent proteins (Henderson & Remington, 2006 ▸). However, fluorescent mutants of chromoproteins can retain *trans* or noncoplanar chromophore conformations, suggesting that a *cis* coplanar conformation is not a pre­requisite for fluorescence (Prescott *et al.*, 2003 ▸).

To date, structures of ∼30 anthozoan fluorescent proteins have been solved, which has been instrumental in their adoption and engineering for synthetic biology. However, only a few naturally occurring chromoproteins have been structurally characterized (Prescott *et al.*, 2003 ▸; Chan *et al.*, 2006 ▸; Wannier & Mayo, 2014 ▸; Chang *et al.*, 2019 ▸; Chiang *et al.*, 2015 ▸; Gurskaya *et al.*, 2001 ▸; Andresen *et al.*, 2005 ▸). Hence, structural and biochemical information on chromoproteins for engineering purposes are largely inferred from investigations of their fluorescent counterparts, which may limit accuracy and successful utilization. Here, we report the structures of four open-source chromoproteins available as BioBricks from the Registry of Standard Biological Parts (http://parts.igem.org): gfasPurple from *Galaxea fascicularis*, amilCP from *Acropora millepora*, spisPink from *Stylophora pistillata* and eforRed from *Echinopora forsaliana*. Of these, eforRed is also fluorescent, despite its high molar extinction coefficient like other chromoproteins (Alieva *et al.*, 2008 ▸). We have detailed the chromophore structures and interacting residues in these proteins, as well as the residues and interactions that are involved in homodimer formation, which seem to be important for successful coloured protein expression.

## 2. Methods

### 2.1. Protein expression

The plasmid constructs for expressing gfasPurple, amilCP, spisPink and eforRed in *E. coli* were purchased from Twist Biosciences (San Francisco, USA). The sequences for the proteins were codon-optimized and placed downstream of a 6×His tag and a TEV protease cleavage site in the expression vector pETcc2, a pET-14b derivative (Peat *et al.*, 2013 ▸). Competent *E. coli* BL21(DE3) cells (New England Biolabs) were transformed and plated on Luria Broth (LB) agar plates containing 100 µg ml^−1^ ampicillin. The plates were incubated overnight at 37°C and a single colony from each plate was inoculated into 10 ml LB medium with 100 µg ml^−1^ ampicillin and grown for 4–6 h with shaking at 200 rev min^−1^ at 37°C. This starter culture was then used to inoculate 1 l auto-induction medium (5 g yeast extract, 20 g tryptone, 85.5 m*M* NaCl, 22 m*M* KH_2_PO_4_, 42 m*M* Na_2_HPO_4_, 0.6% glycerol, 0.05% glucose and 0.2% lactose) with 100 µg ml^−1^ ampicillin, and the culture was grown overnight at 30°C with shaking at 200 rev min^−1^. Cells were harvested the next day by centrifugation at 4000*g* for 10 min at 4°C and frozen at −20°C until purification.

### 2.2. Protein purification

Cells were resuspended in lysis buffer (50 m*M* Tris, 300 m*M* NaCl pH 7 for spisPink and pH 8 for the other proteins) and lysed using a homogeniser (M-110P Microfluidizer, Microfluidics). The lysate was then spun at 20 000*g* for 30 min at 4°C. The resulting supernatant was loaded onto a 5 ml Fast Flow Ni–NTA column (Cytiva). The loaded column was washed with 20 ml lysis buffer followed by 20 ml lysis buffer with 10% buffer *B* (50 m*M* Tris, 300 m*M* NaCl, 500 m*M* imidazole pH 7 for spisPink and pH 8 for the other proteins). Proteins were then eluted with 20 ml 100% buffer *B*. 15 ml of the eluted protein was loaded onto a HiPrep 26/10 desalting column (Cytiva) and eluted with a buffer consisting of 50 m*M* Tris, 150 m*M* NaCl, 0.5 m*M* EDTA, 1 m*M* DTT, 1% glycerol. The remaining eluent from the Ni–NTA column was aliquoted into 500 µl tubes, flash-frozen in liquid nitrogen and stored at −80°C. In-house-purified TEV protease was added to the desalted protein (1 mg TEV protease per 10 mg chromo­protein) and incubated overnight at room temperature. The cleaved His tag was removed by passing the mixture through the Ni–NTA column again; the cleaved protein was collected in the flowthrough. The sample was then concentrated to ∼1 ml using a 15 ml 10 kDa molecular-weight cutoff concentrator (Millipore) and further purified by size-exclusion chromatography using a HiLoad 16/600 Superdex 200 pg column (Cytivia) with a running buffer consisting of 20 m*M* HEPES, 50 m*M* NaCl pH 7.5. Finally, the samples were concentrated to ∼1 m*M* for crystallization using a 15 ml 10 kDa molecular-weight cutoff concentrator (Millipore).

### 2.3. Crystallography

The four proteins (in the gel-filtration buffer 20 m*M* HEPES, 50 m*M* NaCl pH 7.5) were set up at the concentrations provided from the purification step. Each protein was set up in four 96-well plates using a standard crystallization screening protocol (Shotgun, PACT and PS_gradient screens at 20°C and Shotgun screen at 8°C). The proteins were set up using a Phoenix dispensing robot (ARI, USA) in 300 nl droplets (150 nl protein solution and 150 nl reservoir solution). The drops were dispensed into SD-2 sitting-drop plates (Swissci, UK) equilibrated against a reservoir of 50 µl. Plates were sealed with UVXPO clear sealing film (Swissci, UK), incubated and inspected in RockImager 1000 imaging systems (Formulatrix, USA).

Crystals of sufficient size and quality were obtained from the initial screens after about a week. The crystals for diffraction experiments were cryoprotected in the drop (by adding glycerol to the reservoir to a final concentration of 15% and then layering 1 µl of the supplemented reservoir solution over the drop) before being harvested into MiTeGen loops and flash-cooled by plunging into liquid nitrogen. All X-ray data were collected at 100 K on beamlines MX1 and MX2 at the Australian Synchrotron as indicated in Supplementary Table S1 (Cowieson *et al.*, 2015 ▸; Aragão *et al.*, 2018 ▸; McPhillips *et al.*, 2002 ▸).

The images were processed with *autoPROC* (Vonrhein *et al.*, 2011 ▸) utilizing *XDS* (Kabsch, 2010 ▸) and the *CCP*4 suite of programs (Winn *et al.*, 2011 ▸). Anisotropic high-resolution limits were applied to the processed reflections using *STARANISO* (Tickle *et al.*, 2018 ▸) and the final resolution cutoff was chosen based on combination of the parameters in the high-resolution shell: CC_1/2_ > 0.5, *I*/σ(*I*) > 1.0, *R*_p.i.m._ < 0.6. The structure of gfasPurple was solved by molecular replacement with *Phaser* (Evans & Murshudov, 2013 ▸) using PDB entry 3ir8 (Henderson *et al.*, 2009 ▸) as the search model, and the structures of the other three proteins were solved using the final gfasPurple model as the molecular-replacement search model. The models were built in *Coot* (Emsley *et al.*, 2010 ▸) and were refined with *autoBUSTER* using noncrystallographic restraints (except for gfasPurple) and TLS (Bricogne *et al.*, 2017 ▸; Smart *et al.*, 2012 ▸). Statistics for processing and refinement can be found in Table 1 ▸. The Ramachandran statistics for each final model are as follows (expressed as percentage favoured/outliers): gfasPurple, 98.58/0.0; amilCP, 99.47/0.0; eforRED, 99.07/0.12; spisPINK, 99.53/0.0.

### 2.4. GfasPurple mutagenesis and screening

The semi-random mutant library of gfasPurple was generated by purchasing a 186 bp fragment library from Twist Biosciences with the following amino-acid substitutions: R149D/E/H/N/Q, R145D/E/H/N/Q, F158A/H/I/L/V, Y190F/H/S/T, Y188D/E and F147H/F. This fragment library was amplified by PCR with Phusion polymerase (using the manufacturer’s protocol with an annealing temperature of 63°C) using the primers 5′-CCAAGGTTGGGAACCGAATAC-3′ and 5′-GGTCACATCCAGTTTACGATCC-3′. The vector backbone was amplified similarly using the gfasPurple construct as a template with the primers 5′-GTGGATCGTAAACTGGATGTGAC-3′ and 5′-TTCGGTATTCGGTTCCCAACC-3′, followed by digestion with DpnI (New England Biolabs) at 37°C for 10 min to remove any template DNA. The purity of the PCR products was analysed by gel electrophoresis and single bands of the correct size were gel-purified (Wizard SV Gel and PCR Clean-up System, Promega). The library was assembled using NEB assembly (New England Biolabs) as per the vendor’s recommendation using a 1:5 backbone:insert molar ratio. 2 µl each of the assembled reactions was transformed into 2 × 50 µl NEB T7 Express *E. coli* cells (New England Biolabs) by heat shock at 42°C for 10 s followed by 5 min on ice. Each cell aliquot was resuspended in 1 ml recovery medium (New England Biolabs) and incubated with shaking for 1 h at 37°C. 100 µl aliquots were plated on 15 LB agar plates containing 100 µg ml^−1^ ampicillin and the plates were incubated at 37°C overnight followed by two days at room temperature before screening for coloured colonies.

To generate the random mutant library, gfasPurple was amplified using Mutazyme II DNA polymerase (GeneMorph II Random Mutagenesis Kit, Agilent) with the primers 5′-GGAGAACCTGTATTTTCAGGGTATG-3′ and 5′-TTATTGCTCAGCGGATCCTTAG-3′. The PCR reaction consisted of 35.5 µl nuclease-free water, 5 µl 10× Mutazyme II buffer, 1 µl 40 m*M* dNTP stock solution, 2.5 µl 10 µ*M* primer solution, 1 µl Mutazyme II and 0.5 µl template plasmid (0.1 ng template DNA). The PCR conditions were 95°C for 2 min, 30 cycles of 95°C for 30 s, 55°C for 30 s and 72°C for 1 min, and finally 72°C for 10 min. The vector backbone was amplified using Phusion polymerase and digested with DpnI as above using the primers 5′-CATACCCTGAAAATACAGGTTCTCC-5′ and 5′-CTAAGGATCCGCTGAGCAATAAC-3′. The fragments were gel-purified (Wizard SV Gel and PCR Clean-up System, Promega) and the library was assembled using NEB assembly (New England Biolabs) as above with a 1:2 backbone:insert molar ratio. The library was then transformed into NEB T7 Express *E. coli* cells (New England Biolabs) and screened in the same way as the semi-random library.

Coloured colonies were picked, re-streaked onto a fresh LB agar plate (with 100 µg ml^−1^ ampicillin) and inoculated into 2 ml auto-induction medium containing 100 µg ml^−1^ ampicillin in a 48-well growth block, which was incubated for two days at 30°C with shaking at 1050 rev min^−1^. Growth blocks containing coloured cultures were spun at 4000*g* for 10 min at 4°C to harvest the cells and were stored at −20°C until further use. The pellets or re-streaked plates were used for colony PCR using Quick-Load Taq 2× Master Mix (New England Biolabs) with standard T7 primers, and the PCR fragments were sent for Sanger sequencing (Macrogen).

The constructs for the single mutants of gfasPurple were assembled in the same way as the random library into the same vector backbone, except that each mutant gene was purchased from IDT (Integrated DNA Technology, Australia) as a gene fragment. After transformation, 100 µl was plated on one plate or 50 µl on half a plate and was incubated at 37°C overnight followed by two days at room temperature.

### 2.5. UV–visible spectroscopy

Cell pellets from 2 ml small-scale cultures were lysed in 400 µl lysis buffer consisting of 50 m*M* Tris, 300 m*M* NaCl pH 8 and 1× BugBuster Protein Extraction Reagent (Millipore) and incubated on ice for 15 min in a 1.5 ml microfuge tube. The tubes were spun at 20 000*g* for 20 min at 4°C and the resulting supernatant was transferred into a clear 96-well plate. UV–visible spectra (300–750 nm) were obtained using a SpectraMax M3 Microplate reader from Molecular Devices. The spectra of purified gfasPurple, amilCP, spisPink and eforRed were obtained similarly, where the samples contained protein eluted from the Ni–NTA column diluted approximately 1:100 in 50 m*M* Tris, 300 m*M* NaCl pH 7.5.

### 2.6. SDS–PAGE

Protein samples were heated at 98°C in sample buffer [NuPAGE LDS Sample Buffer (4×), Invitrogen] for 8 min and were loaded onto a pre-cast NuPAGE 4–12% Bis-Tris gel (Invitrogen), which was run for 30–40 min at 150 V in MES SDS running buffer (Invitrogen). Gels were stained with AcquaStain Protein Gel Stain (Bulldog) for 30 min and destained in water.

## 3. Results and discussion

### 3.1. Protein production, purification and spectral analysis

**Figure 1:**
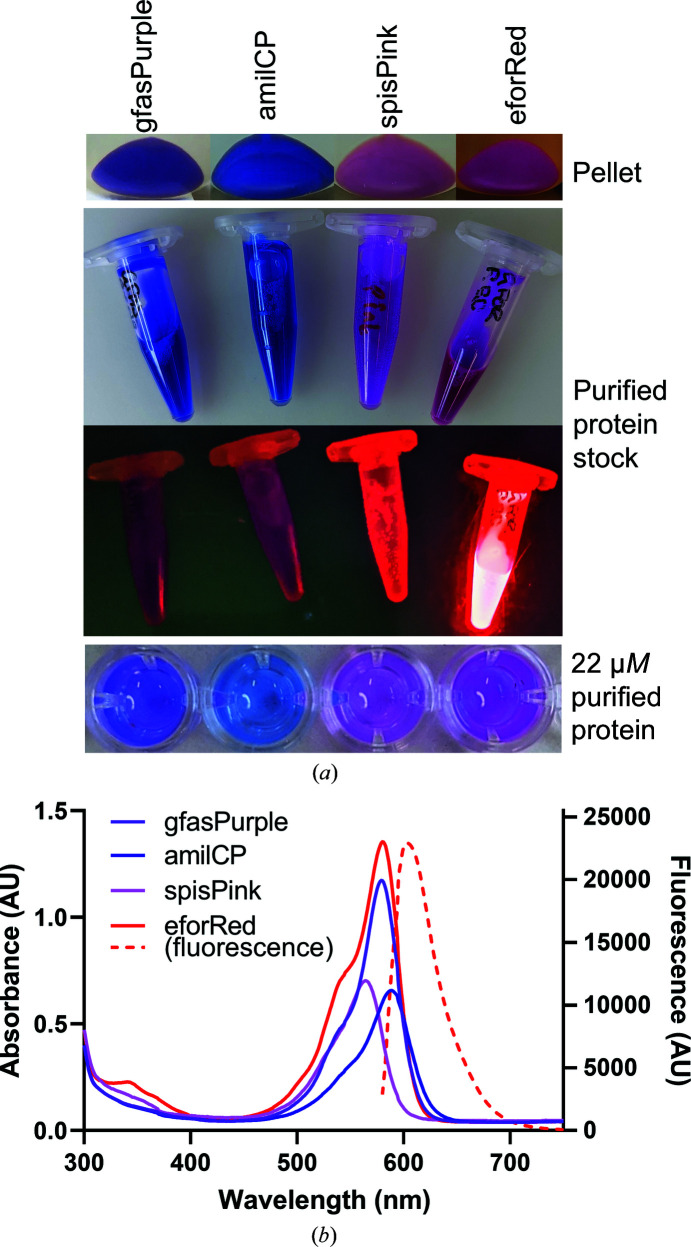
(*a*) Colours of the chromoproteins purified in this study. The purified protein stocks (concentration >1 m*M*) are shown in both ambient light and under a blue-light filter on a blue-light box. (*b*) Absorbance and fluorescence spectra of each chromoprotein at 22 µ*M* in buffer containing 50 m*M* Tris and 300 m*M* NaCl at pH 7.5. The fluorescence spectrum is only shown for eforRed as the others did not have any detectable peaks.

The proteins were purified using Ni–NTA protein chromatography and the visible colours of the purified proteins are as follows: purple for gfasPurple, indigo-blue for amilCP, pink for spisPink and pink-red or rose for eforRed (Fig. 1 ▸ *a*). Only eforRed demonstrated visible fluorescence under blue light (Fig. 1 ▸ *a*) or when excited at the absorbance maximum (Fig. 1 ▸ *b*). The absorbance spectra revealed similar absorbance maxima for gfasPurple and eforRed (579 and 580 nm, respectively) despite their different visible colours. The reddish appearance of eforRed can be attributed to its intense, red-shifted fluorescence emission that peaks at 602 nm. The absorbance maxima for amilCP and spisPink are less intense, with a blue shift in spisPink to 564 nm and a red shift in amilCP to 588 nm.

### 3.2. GfasPurple, amilCP, spisPink and eforRed are dimers in solution

**Figure 2:**
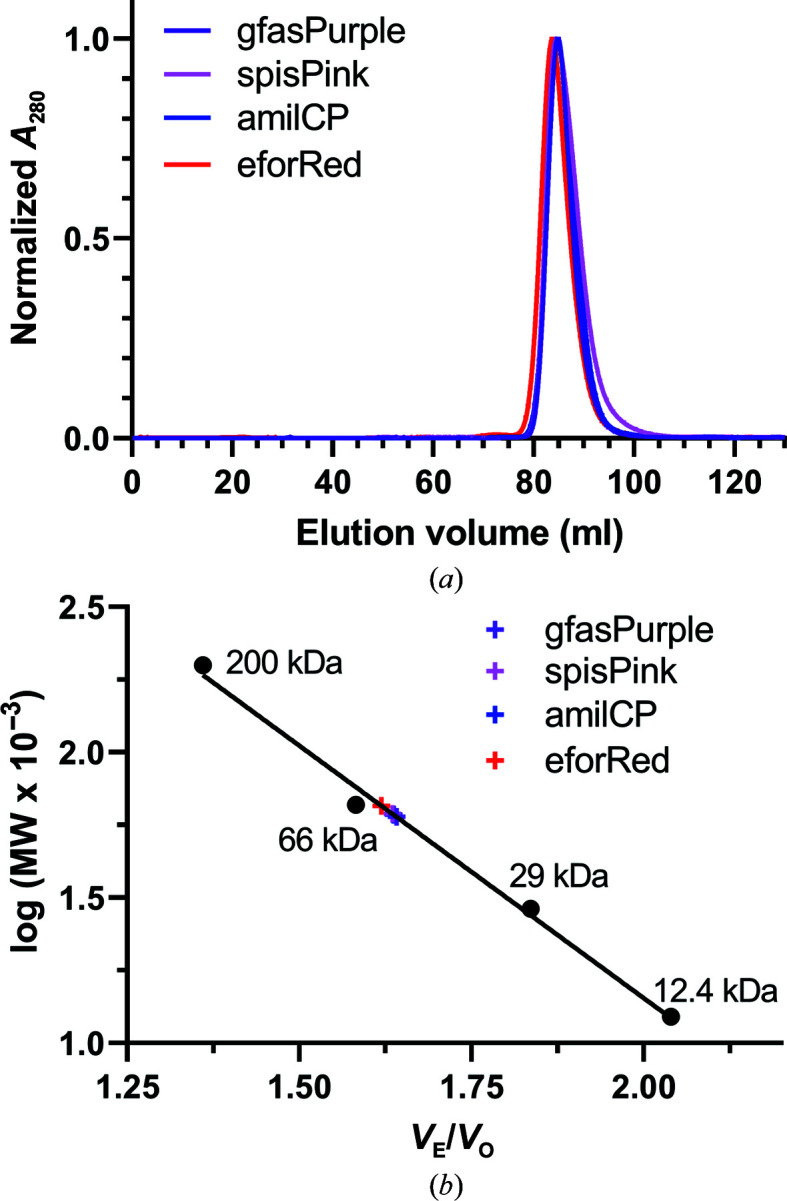
Size-exclusion chromatography of gfasPurple, spisPink, amilCP and eforRed. (*a*) Normalized elution profiles for the proteins showing their elution as single peaks at ∼85 ml. (*b*) Comparison of the retention of the chromoproteins [elution volume (*V*_E_)/void volume (*V*_O_)] with protein standards. The interpolated estimated mass is marked for each protein.

The proteins were then further purified and characterized by size-exclusion chromatography. Elution volumes of ∼85 ml were obtained for all four proteins on a preparative-grade 120 ml Superdex 200 pg column (Fig. 2 ▸ *a*), which correspond to a molecular mass of ∼60 kDa (Fig. 1 ▸ *b*). The theoretical masses of the monomeric proteins are 24.9 kDa for gfasPurple, 24.9 kDa for spisPink, 25.0 kDa for amilCP and 25.6 kDa for eforRed, suggesting that the proteins were eluting as dimers and not as tetramers of ∼100 kDa. Other naturally occurring chromoproteins such as shCP (Chang *et al.*, 2019 ▸) and sgBP (Chiang *et al.*, 2015 ▸) have also been shown to be dimers in solution, although many exist as tetramers, for example Rtms5 (Pettikiriarachchi *et al.*, 2012 ▸), asFP595 (Andresen *et al.*, 2005 ▸) and hcCP (Gurskaya *et al.*, 2001 ▸), as well as the related fluorescent proteins dsRed (Gross *et al.*, 2000 ▸) and Azami-Green (Karasawa *et al.*, 2003 ▸). Oligomerization of naturally occurring GFP-like proteins is well documented. In these proteins, homodimerization tends to occur at an unusually polar interface with an extensive hydrogen-bonding network incorporating water molecules, and tetramerization occurs when two dimers associate at a second interface formed by an area of hydrophobic residues surrounded by polar residues (Chang *et al.*, 2019 ▸; Wall *et al.*, 2000 ▸).

### 3.3. Structures of gfasPurple, amilCP, spisPink and eforRed

All of the proteins crystallized very readily, both quickly (starting within minutes of being set up) and in many different crystallization conditions, particularly those containing mid-weight polyethylene glycols (Supplementary Fig. S1). The crystallization conditions of the crystals that were used in X-ray data collection are shown in Supplementary Table S1. The data sets collected were to resolutions of 1.39 Å in space group *P*4_2_22 for gfasPurple, 1.64 Å in space group *P*12_1_1 for amilCP, 2.01 Å in space group *P*2_1_2_1_2_1_ for eforRed and 1.44 Å in space group *P*12_1_1 for spisPink (Table 1 ▸).

**Figure 3:**
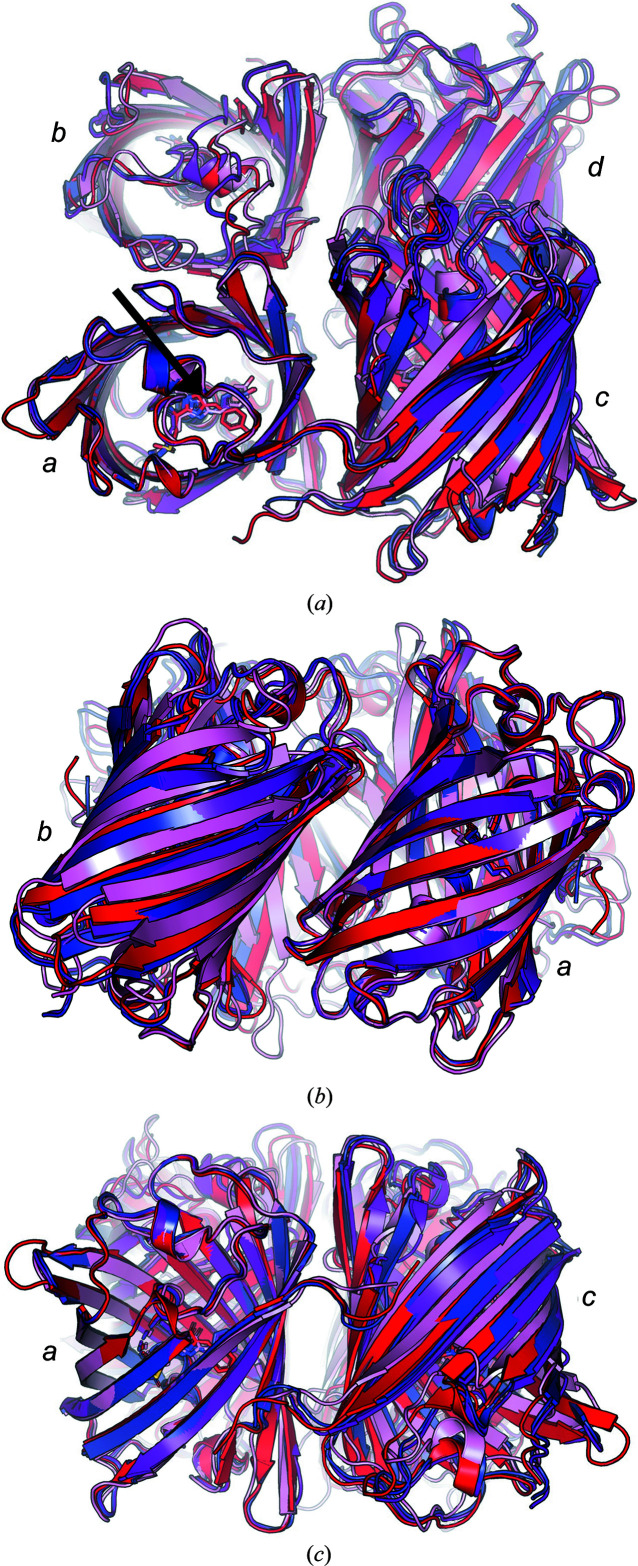
Topology of the structures of gfasPurple (purple), amilCP (blue), eforRed (red) and spisPink (pink). (*a*) Top-down view of the tetrameric arrangement of chromoprotein monomers. The location of the chromophore (stick representation) is marked with a black arrow in molecule *a*. All of the molecules in the asymmetric unit are shown for eforRed and spisPink, while only four molecules in a tetrameric arrangement are shown for amilCP. Crystallographic symmetry mates are shown for the single gfasPurple molecule present in the asymmetric unit. Molecule annotations (*a*, *b*, *c* or *d*) are for illustration only and may not align with chain IDs in the PDB file. (*b*) Side view showing the *a*/*b* interface and (*c*) side view showing the *a*/*c* interface.

Like other chromoproteins and fluorescent proteins, each chain of gfasPurple, amilCP, eforRed and spisPink folds into a β-barrel consisting of 11 β-strands (Fig. 3 ▸ *a*). A partially formed α-helix passes through the barrel, with the autocatalytically formed chromophore located at the centre. The average pairwise r.m.s.d. between the monomers of the four proteins is 0.62 Å (as calculated with *mTM-align*; Dong *et al.*, 2018 ▸), suggesting that the overall protein topology is conserved between them.

**Figure 4:**
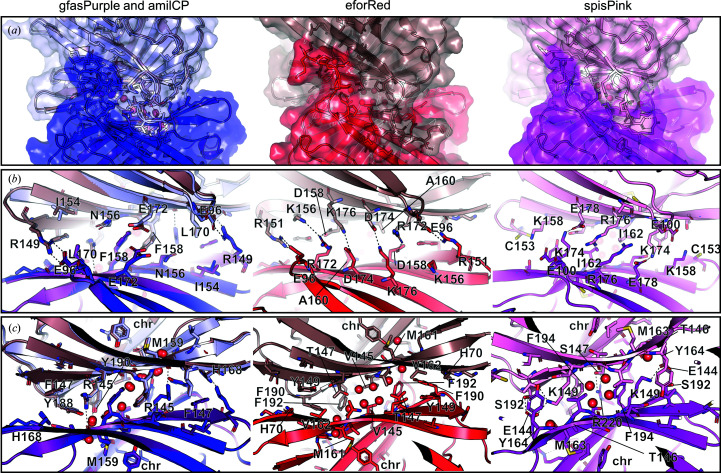
The *a*/*c* dimer interface. GfasPurple (purple) and amilCP (blue) are overlapped and shown as one panel due to their high sequence identity (98.2%). For all four proteins, the secondary chain is shown in a lighter colour. Dashed lines represent salt bridges or hydrogen bonds. (*a*) Surface representation of proteins showing the C-termini of the chains in the homodimer forming a symmetrical ‘embrace’. For gfasPurple and amilCP, the surface of amilCP is shown. (*b*) Interactions at the dimer interface viewed from the opposite surface to the C-termini shown in (*a*). (*c*) A detailed view into deeper interfacing interactions in the same plane as in (*b*). Water molecules forming a solvent channel are shown as red spheres and the chromophore is labelled ‘chr’. Residues are labelled in either (*b*) or (*c*), where they may or may not be visible in both panels.

The structures of eforRed and spisPink contain four molecules in the asymmetric unit, arranged as typically reported for tetrameric members of this protein family (Fig. 3 ▸ *a*). The structure of amilCP has eight molecules in the asymmetric unit, with four of them in the same tetrameric arrangement and the other four forming a second tetramer within crystallographic symmetry. GfasPurple has only one molecule in the asymmetric unit, but its crystallographic symmetry expansion also revealed a similar tetrameric arrangement of monomers. In this arrangement, each molecule interacts with two others, creating either an *a*/*b* or an *a*/*c* interface (Figs. 3 ▸ *b* and 3 ▸ *c*). Both interfaces demonstrate a twofold horizontal rotational symmetry, where symmetrical residues from each monomer form interactions (Fig. 4 ▸).

### 3.4. Interactions at the dimer interface

To elucidate which protein interface is most likely to form the dimeric oligomerization observed in solution (Fig. 2 ▸), we analysed these four structures using the *PDBePISA* interactive tool (Krissinel, 2010 ▸). This tool calculates the chemical properties of protein interfaces and assesses their significance to help identify biologically relevant complexes (Supplementary Table S2). The data obtained suggested that the larger *a*/*c* interface, with surface areas of between 1061 and 1368 Å^2^, is more likely to be relevant for complex formation compared with the *a*/*b* interface (792–1006 Å^2^). However, the algorithm only gives decisive results for spisPink and eforRed, and predicted just a twofold higher score for the *a*/*c* interface in gfasPurple and amilCP, which have 98.2% sequence identity. This suggests that oligomerization of the latter proteins could be context-dependent, depending for instance on buffer conditions or temperature. Indeed, the closely related protein Rtms5, which has a sequence identity of 95.5% to amilCP and of 94.6% to gfasPurple, has been shown to be tetrameric in solution (Pettikiriarachchi *et al.*, 2012 ▸).

A detailed inspection revealed that the *a*/*b* interface of all four proteins primarily involves polar interactions, with the same threonine residues in both chains hydrogen-bonding to each other at the centre (Supplementary Fig. S2). In contrast, the *a*/*c* interface is more complex and involves the C-termini of the proteins, multiple salt bridges, hydrogen bonds and hydrophobic interactions (Fig. 4 ▸). On one side, the C-termini of both chains form a tight ‘embrace’ (Fig. 4 ▸ *a*) which is facilitated by polar interactions with the opposite chain. This is similar to the related fluorescent proteins hcRed (Wannier *et al.*, 2018 ▸) and dsRed (Wall *et al.*, 2000 ▸; Campbell *et al.*, 2002 ▸), where deletion or extensive mutation of the C-termini was instrumental in breaking the *a*/*c* interface to form monomeric variants. The opposite side of the interface is held predominantly by symmetrical salt bridges formed between conserved ionic residues contributed by both chains (Fig. 4 ▸ *b*, Supplementary Fig. S3). These are most extensive in eforRed, involving six main interactions: four salt bridges between two sets of Glu96–Arg151 and Asp154–Lys176 residues and two hydrogen bonds between Arg172 and Lys176. In contrast, only one set of these interactions are observed in spisPink (Lys174–Glu178), gfasPurple (Glu96–Arg149) and amilCP (Glu96–Arg149).

Interestingly, interactions deeper within the *a*/*c* interface do not appear to be as specific as the salt bridges and polar interactions observed at the edges, although there is still some degree of conservation between the four proteins (Fig. 4 ▸ *c* and Supplementary Fig. S2). The same hydrophobic residue from each chain is at the very centre; the Phe158 residues in gfasPurple and amilCP form π–π interactions with each other, while the two Ala190 and Ile162 residues in eforRed and spisPink, respectively, form hydrophobic interactions. Nearby, Arg145 (in gfasPurple and amilCP), Thr147 (in eforRed) and Lys149 (in spisPink) form polar or charged interactions with the opposite chain (Fig. 4 ▸ *c*). In eforRed, this interaction is mediated through a water molecule, which is part of a solvent channel that links the conserved chromophore-interacting Met161 in both chains (Fig. 4 ▸ *c*, Supplementary Fig. S3). Similar water channels are present in gfasPurple and amilCP, stretching between the chromophore-interacting Met159 residues. In these two proteins, the water molecules also hydrogen-bond to four tyrosine residues (Tyr188 and Tyr190), as well as the protein backbone, aiding the stability of the remaining imperfectly packed hydrophobic residues within the interface. In spisPink, two waters are replaced by Thr146 from each monomer, and hence the hydrogen-bonding network linking the two chromophores *via* the conserved methionine residue (Met163) is still maintained.

The *a*/*c* interface of these proteins demonstrates a nonpolar central region surrounded by polar interactions and salt bridges, similar to previous observations for the chromo­protein Rtms5 (Prescott *et al.*, 2003 ▸). This is fairly typical for protein–protein interfaces in homodimers, although usually the hydrophobic residues at the centre are packed to fully exclude solvent (Bahadur *et al.*, 2003 ▸). Interestingly, a similar solvent channel linking the equivalent lysine residues of both chains is also present in the tetrameric structure of the related fluorescent protein dsRed, although it instead contains multiple polar and charged residues at the centre of the interface (Wall *et al.*, 2000 ▸). Studies of the monomerized dsRed variants mCherry and mOrange indicate that the stabilization of the corresponding position of the conserved methionine in these chromoproteins, by mutation to glutamine for increased rigidity or mutating surrounding residues, enhances the photostability that is usually lost upon monomerization (Regmi *et al.*, 2013 ▸; Shaner *et al.*, 2008 ▸). Hence, it is possible that the hydrogen-bonding network created by the water molecules due to oligomerization has a role in maintaining the conformation of this conserved methionine residue to aid chromophore maturation or stability.

### 3.5. Mutation at the dimer interface of gfasPurple leads to loss of colour

To probe whether dimerization is important for coloured protein expression, we tried disrupting the *a*/*c* dimer interface of gfasPurple by generating a semi-random mutant library to prevent inter-chain interactions (R149D/E/H/N/Q, R145D/E/H/N/Q, F158A/H/I/L/V, Y190F/H/S/T, Y188D/E and F147H/F). These residues form a salt bridge (Arg149), hydrogen bonds (Arg145, Tyr190) or hydrophobic interactions (Phe158, Phe147 and Tyr188) with the secondary chain at the *a*/*c* dimer interface (Figs. 4 ▸ *b* and 4 ▸ *c*), and the mutations were selected to disrupt these interactions while maintaining a relatively small mutant library size of 2000 variants. However, all coloured colonies from the screen had the original sequence without mutation, and no colonies with varying shades of purple were observed.

For further investigation, we generated a high-frequency random mutant library with a potential diversity of 2400 variants (average mutation frequency of 4.5 nucleotide substitutions per variant) and sequenced any coloured colonies obtained. From the 2000 colonies screened, only 5% demonstrated any colour, and of these 25% possessed genes with the original sequence or that carried only synonymous mutations. The remaining 75% included one colony with a visibly more bluish shade of purple compared with gfasPurple and two colonies that were pink, while the rest demonstrated various intensities of the same purple colour as gfasPurple. On average, the sequences of coloured variants contained two amino-acid substitutions, with the highest number of substitutions being four. The colourless mutants contained an average of four amino-acid substitutions, and 40% of them had acquired premature STOP codons. Analysis of the sequences of the coloured mutants revealed fewer mutations at the *a*/*c* interface compared with the rest of the protein surface (Supplementary Fig. S4). Amino-acid substitutions tolerated in the interface were F158Y, R145C and H168D, all of which had noticeably reduced pigmentation in cell pellets compared with the wild-type protein. Substitutions were also found at the C-terminus (Lys217, Ser218 and Val219) in residues that do not interact with the opposite chain. Finally, we also made single amino-acid substitutions to disrupt salt-bridge formation (E96D, R149H and R149K), anion–π interactions (E140D) and hydrophobic interactions (F158V) at the *a*/*c* interface, with the intent to minimize changes to the surface character. These five mutants also produced colourless colonies (Supplementary Fig. S4).

These results led us to hypothesize that dimerization at the *a*/*c* interface may be necessary for pigmentation in native gfasPurple, at least without a simultaneous compensating mutation elsewhere to enhance the stability of the monomeric form. Only one chromoprotein, Rtms5, has been monomerized to date with colour retention (Pettikiriarachchi *et al.*, 2012 ▸). Rtms5 is a tetramer and has 94.6% sequence identity to gfasPurple. In this protein, two mutations had to be introduced at the chromophore-binding site to restore colour to a colourless monomeric variant with mutations to the dimeric interface. Interestingly, mutation of these same positions in gfasPurple (S125R, F162R and V44A with or without L123T) failed to produce a coloured variant (Supplementary Fig. S4), suggesting that the monomerizing mutations are not necessarily transferrable between closely related chromoproteins. Generating monomeric variants of the anthozoan red fluorescent proteins dsRed and hcRed also required intensive mutation and optimization, including 13 and 11 mutations in close proximity to the chromophore, respectively, in addition to multiple mutations at the interfaces (Wannier *et al.*, 2018 ▸; Campbell *et al.*, 2002 ▸).

### 3.6. No *N*-acylimine formation in the chromophore of spisPink

**Figure 5:**
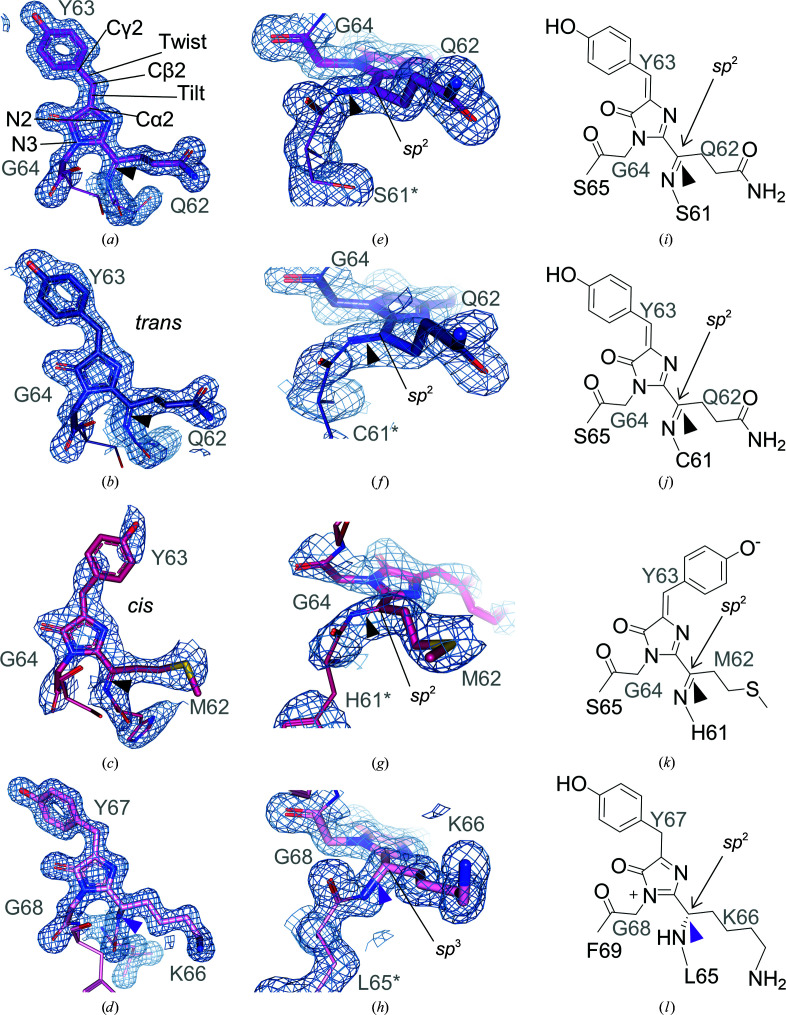
The chromophores of gfasPurple (purple), amilCP (dark blue), eforRed (red) and spisPink (pink). Residues forming the chromophores are labelled in grey. (*a*)–(*d*) Composite omit maps (2*mF*_o_ − *DF*_c_) contoured at 1.5σ showing the electron density for the chromophores (sticks) and the preceding residue in the amino-acid chain (lines). The subsequent amino acid is also shown as lines. Atoms and bonds discussed in the text are labelled in the chromophore for gfasPurple, and *trans* and *cis* chromophore conformations are labelled for amilCP and eforRed. (*e*)–(*h*) Side view showing the peptide link between the chromophore and the preceding amino acid (labelled in grey with *), suggesting *N*-acylimine formation (black arrowhead) in gfasPurple, amilCP and eforRed but not in spisPink (purple arrowhead). (*i*)–(*l*) Chemical structures of the chromophores in gfasPurple, amilCP, eforRed and spisPink, respectively, inferred from the electron density. The ionized form is shown for eforRed as in other related red fluorescent proteins (Miyawaki *et al.*, 2012 ▸), and *N*-acylimine formation is highlighted in the same way as in (*e*)–(*h*).

The structures obtained for all four proteins are at high resolution (ranging from 1.4 to 2 Å) and are well defined, showing clear electron density for the chromophores (Figs. 5 ▸ *a*–5 ▸ *d*). GfasPurple and amilCP have the same chromophore composition (Gln62-Tyr63-Gly64), while the chromophore is composed of Met62-Tyr63-Gly64 in eforRed and Lys66-Tyr67-Gly68 in spisPink.

The electron densities for gfasPurple and amilCP suggest that the Gln62 moiety of the chromophore is *sp* ^2^-hybridized and planar to form an *N*-acylimine (C=N) and is linked to the preceding Ser61 or Cys61, respectively, by a *cis*-peptide bond (Figs. 5 ▸ *e*, 5 ▸ *f*, 5 ▸ *i* and 5 ▸ *j*). This is similar to the chromoproteins Rtms5 (Chan *et al.*, 2006 ▸) and sgBP (Chiang *et al.*, 2015 ▸), as well as the fluorescent protein dsRed (Gross *et al.*, 2000 ▸), which all contain the same QYG chromophore. *N*-acylimine formation was confirmed by boiling protein samples, which cleaves the *N*-acylimine bond as it is unstable and prone to hydrolysis once the protein has been denatured. Analysis of these samples on an SDS–PAGE gel revealed the presence of three bands in gfasPurple and amilCP: the full-length protein at ∼27 kDa, the N-terminal fragment at ∼9 kDa and the C-terminal fragment at ∼18 kDa (Supplementary Fig. S5).

Boiled eforRed also demonstrated three bands on the SDS–PAGE gel (Supplementary Fig. S5), suggesting *sp* ^2^ hybridization and *N*-acylimine formation at the equivalent Met62 moiety of its chromophore (Figs. 5 ▸ *g* and 5 ▸ *k*). This is similar to all previously structurally characterized anthozoan red fluorescent proteins, which show *N*-acylimine formation regardless of the amino-acid composition of their chromophores (Chan *et al.*, 2006 ▸; Wannier & Mayo, 2014 ▸; Chang *et al.*, 2019 ▸; Chiang *et al.*, 2015 ▸; Gurskaya *et al.*, 2001 ▸; Andresen *et al.*, 2005 ▸; Henderson & Remington, 2006 ▸). In these proteins, extension of π-conjugation to the *N*-acylimine is essential to generate their far-red fluorescence (Gross *et al.*, 2000 ▸). A far-red fluorescent mutant of the chromoprotein aeCP597 that can either mature into a red or green form and has the same MYG chromophore as eforRed contains a mixture of proteins with *N*-acylimine and *N*-acylamine formation (Wannier & Mayo, 2014 ▸). In aeCP597, *N*-acylimine formation was attributed to the red chromophore (Wannier & Mayo, 2014 ▸).

In contrast, Lys66 that forms the corresponding chromophore moiety in spisPink shows clears density for *sp* ^3^ hybridization (*N*-acylamine) with a true *trans*-peptide bond linking the preceding amino acid in the protein chain (Figs. 5 ▸ *h* and 5 ▸ *l*). There is also a notable absence of two clear bands on the SDS–PAGE gel that would result from the hydrolysis of an *N*-acylimine bond in the chromophore (Supplementary Fig. S5). We believe that this is the first observed instance of a chromoprotein chromophore without *N*-acylimine formation. A similar *sp* ^3^ hybridization in this moiety is observed in the chromophores of blue and green fluorescent proteins, which are formed from an alternative route in the branched pathway of the chromophore maturation process compared with ‘dsRed-like’ red fluorescent proteins such as eforRed (Miyawaki *et al.*, 2012 ▸; Strack *et al.*, 2010 ▸; Stepanenko *et al.*, 2011 ▸). In phylogenetic analyses of anthozoan chromoproteins and related fluorescent proteins that have a common ancestor, gfasPurple, amilCP and eforRED are within the same larger clade with ‘dsRed-like’ red fluorescent proteins, whereas spisPink belongs to a sister clade containing predominantly blue and green fluorescent proteins (Alieva *et al.*, 2008 ▸; Lapshin *et al.*, 2015 ▸). The closest structurally characterized protein to spisPink is the blue fluorescent protein amFP486 (53% sequence identity), which has the same KYG chromophore with *sp* ^3^ hybridization at this position (Henderson & Remington, 2005 ▸).

### 3.7. Chromophores adopt noncoplanar conformations

**Figure 6:**
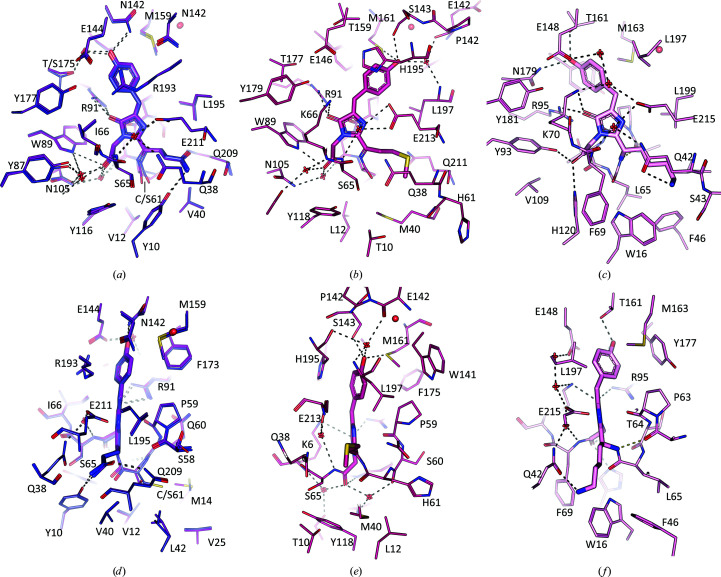
Ligand-binding sites of gfasPurple (purple), amilCP (dark blue), eforRed (red) and spisPink (pink). GfasPurple and amilCP are overlapped and shown together due to their high sequence identity (98.2%). (*a*)–(*c*) Front view showing chromophore-binding interactions. Hydrogen bonds are indicated by dashed black lines and the water molecule near the conserved methionine residue (Met159/Met161/Met163), which is part of the hydrogen-bonding network linking the two dimers shown in Fig. 4 ▸(*c*), is shown as a sphere. (*d*, *e*) The same as (*a*)–(*c*) but in a side view parallel to the plane of the imidazolinone ring showing the overall planarity of the chromophore.

The tyrosyl moieties of the chromophores in gfasPurple, amilCP and spisPink adopt a *trans* conformation with respect to the glutamyl or lysyl moieties (Fig. 5 ▸ *a*, 5 ▸ *b* and 5 ▸ *d*) and are noncoplanar with respect to the imidazolinone moiety (Figs. 6 ▸ *d* and 6 ▸ *f*). Similar *trans* noncoplanar conformations were also observed in all previously structurally characterized chromoproteins (Chan *et al.*, 2006 ▸; Wannier & Mayo, 2014 ▸; Chang *et al.*, 2019 ▸; Chiang *et al.*, 2015 ▸; Gurskaya *et al.*, 2001 ▸; Andresen *et al.*, 2005 ▸; Henderson & Remington, 2006 ▸). Deviation from coplanarity is achieved by ‘tilting’ about the C^α2^—C^β2^ bond and ‘twisting’ about the C^β2^—C^γ2^ bond (Fig. 5 ▸ *a*).

Despite the identical chemical compositions of their chromophores, amilCP demonstrates a similar ‘tilting’ but is more ‘twisted’ (177.2° and 34.9°, respectively) compared with gfasPurple (177.6° and 25.7°, respectively), which may contribute to their different absorbance maxima (Fig. 1 ▸ *b*) and different molar extinction coefficients (205 200 *M* ^−1^ cm^−1^ for gfasPurple and 87 600 *M* ^−1^ cm^−1^ for amilCP; Alieva *et al.*, 2008 ▸). Their chromophore environments show identical residues at the binding site, except for position 175 that binds the hydroxyl group of the tyrosyl moiety via a bridging water molecule and position 61 that links the glutamyl moiety to the protein chain (Fig. 6 ▸ *a*). Previous work has confirmed that S175T and S61C mutations alone are sufficient to shift the colour of gfasPurple to be the same as that of amilCP (Alieva *et al.*, 2008 ▸).

In comparison, the tyrosyl moiety of the chromophore is more ‘tilted’ (153°) in spisPink and is ‘twisted’ in the opposite direction (−36.8°), resulting in a more out-of-plane conformation with respect to the imidazolinone ring (Figs. 6 ▸ *c* and 6 ▸ *f*). This allows the tyrosyl moiety in spisPink to directly interact with Asn197, whereas the equivalent interaction in amilCP and gfasPurple with Ser175 is mediated through a water molecule (Fig. 6 ▸ *a*).

In contrast to the three nonfluorescent proteins, eforRed adopts a *cis* conformation (Figs. 5 ▸ *c* and 6 ▸ *b*) similar to other characterized anthozoan red fluorescent proteins such as hcRed (Wannier *et al.*, 2018 ▸) and dsRed (Wall *et al.*, 2000 ▸). Direct comparison with dsRed highlights the deviation from coplanarity in eforRed (Fig. 6 ▸ *e* and Supplementary Fig. S6), with a ‘tilt’ of 18.0° and a ‘twist’ of 7.7°, which may explain its lower quantum yield. Studies of photo-switchable fluorescent proteins indicate that coplanarity is more essential for fluorescence than a *cis* chromophore conformation, as fluorescence is also observed in mutants containing *trans* but coplanar chromophore variants (Henderson & Remington, 2006 ▸). The other requirement in fluorescent chromoprotein variants is thought to be a histidine (His197) π-stacking with the tyrosyl moiety (Henderson & Remington, 2006 ▸), which is observed in eforRed but not dsRed (Fig. 6 ▸ *b* and Supplementary Fig. S6). This position is mutated to arginine (Arg193) in amilCP and gfasPurple, which may contribute the lack of fluorescence in these proteins.

### 3.8. Conservation of chromophore interactions in chromoproteins

Despite the different chromophore conformation in eforRed compared with gfasPurple and amilCP, key protein interactions appear to be conserved around the imidazolinone and glutamyl/methionyl moieties (Figs. 6 ▸ *a*–6 ▸ *c*). In all three proteins N3 of the imidazolinone ring is linked to the protein chain by a *trans*-peptide bond to a serine residue (Ser65). The glutamyl moieties in amilCP and gfasPurple are sandwiched between two glutamine residues (Gln38 and Gln209), and this is also the case for the methionyl moiety in eforRed (Gln38 and Gln211). The polar and hydrophobic natures of the two glutamyl and methionyl moieties, respectively, are then complemented by hydrogen bonding to Tyr10 or van der Waals interactions with Met40, respectively. In these three proteins, a glutamate residue (Glu211 or Glu213) hydrogen-bonds to a water molecule suspended above the imidazolinone ring, and the carbonyl on the ring is hydrogen-bonded to an arginine residue (Arg91). The glutamate residue (Glu211 or Glu213) also hydrogen-bonds to N2 of the imidazolinone ring.

At the tyrosyl moiety, the interactions are different for eforRed due to the *cis* conformation of the chromophore (Fig. 6 ▸). Most notably, an unusual hydroxyl-to-methionine hydrogen bond was observed in eforRed between the tyrosyl moiety and Met161, while the equivalent Met159 in gfasPurple and amilCP only forms van der Waals interactions. In spisPink, the corresponding Met163 residue also forms van der Waals interactions with the tyrosyl moiety, aiding its more twisted conformation. The water molecule adjacent to this conserved methionine links the chromophore-binding sites of the two protein chains in the homodimer through a network of water molecules, while also contributing to the hydrogen-bonding network that holds the chromophore. This suggests a role for this intra-dimer hydrogen-bonding network in chromophore binding, which may be an underlying reason for the importance of dimer formation for colour development and fluorescence.

Of the four structures described in this work, the overall chromophore-binding site in spisPink is the most divergent from the others. However, it too has glutamine and glutamate residues (Gln42 and Glu215) that stabilize a water molecule over the imidazolinone ring, with N2 and the carbonyl O atom of the imidazolinone ring hydrogen-bonded to a glutamate residue (Glu215) and an arginine residue (Arg95), respectively (Figs. 6 ▸ *a*–6 ▸ *c*). The most striking difference is that Ser65 in gfasPurple, amilCP and eforRed, which forms a *trans*-peptide bond with N3 of the imidazolinone ring, is replaced by Phe69, adding considerable steric bulk in this region. This is increased by the presence of the nearby Trp16 and His120, the latter hydrogen-bonding to the carbonyl group of the Phe69–chromophore peptide bond. The increased steric interactions displace the imidazolinone ring in spisPink relative to the same position in other chromoproteins (Supplementary Fig. S7), promoting the unusual twist in the tyrosyl moiety for its accommodation in the consequently smaller cavity.

### 3.9. Blue- and red-shifted gfasPurple mutants are like spisPink and amilCP

**Figure 7:**
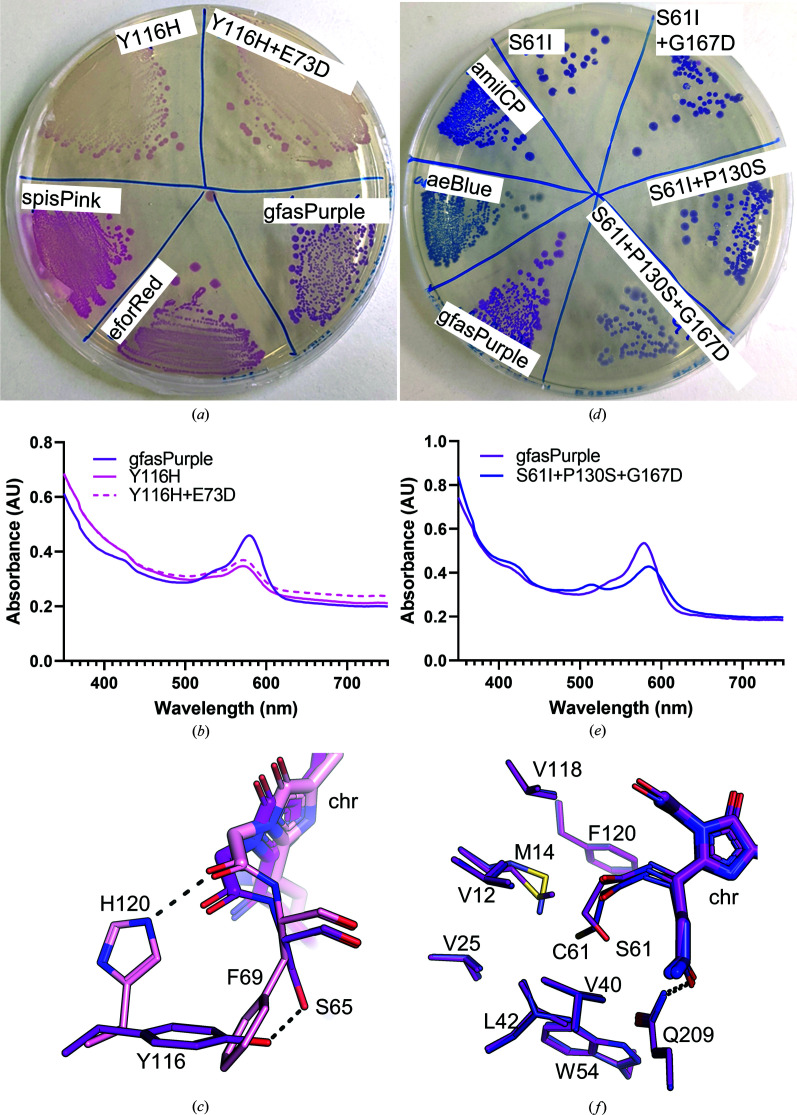
GfasPurple mutants with blue- and red-shifted absorbance spectra. (*a*) Comparison of *E. coli* colonies expressing the Y116H and Y116H+E73D mutants of gfasPurple compared with gfasPurple, spisPink and eforRed. (*b*) Absorbance spectra of cell lysates of *E. coli* expressing the Y116H and Y116H+E73D mutants and gfasPurple. (*c*) Close-up of the chromophore-binding sites of gfasPurple (purple) and spisPink (pink) showing the role of His120 in spisPink and Tyr116 in gfasPurple. (*d*) Comparison of *E. coli* colonies expressing gfasPurple with S61I, S61I+P130S, S61I+G167D and S61I+P130S+G167D mutations with wild-type gfasPurple, amilCP and aeBlue. (*e*) Absorbance spectra of cell lysates of *E. coli* expressing the S61I+P130S+G167D mutant and gfasPurple. (*f*) Close-up of the chromophore-binding sites of gfasPurple (purple) and amilCP (blue) showing the substitution of Ser61 with isoleucine in amilCP.

Our random mutant library of gfasPurple also provides some insight into the chromophore environment of these chromoproteins. We found that coloured variants with mutations of residues that face into the β-barrel were much rarer than those on the protein surface, and all but two of these mutated residues (Tyr116 and Ser761) do not interact directly with the chromophore (Supplementary Fig. S4). The Y116H and S61I mutations obtained also changed the colour of gfasPurple to pink and indigo-purple, respectively (Fig. 7 ▸).

The Y116H mutation was found in two pink variants, which were visibly similar in colour to spisPink (Fig. 7 ▸ *a*). The absorbance maxima for these mutants were blue-shifted to 572 nm compared with 579 nm in gfasPurple (Fig. 7 ▸ *b*), but were not completely shifted to the absorbance maximum of 564 nm seen in spisPink. Sanger sequencing revealed that one of them was the single Y116H mutant and the other contained Y116H and E73D, confirming that the Y116H substitution is responsible for this colour change. Interestingly, the corresponding position to Tyr116 in gfasPurple is His120 in spisPink (Fig. 7 ▸ *c*), suggesting that chromophore interactions by histidine in this position may be important for pink pigmentation. Whether the histidine mutation causes an unusual chromophore twist in gfasPurple to mimic spisPink or just introduces new hydrogen-bonding interactions to the chromophore remains to be investigated.

The S61I mutation that causes a colour change to bluish-violet was first observed in a variant containing the mutations S61I, P130S and G167D (Fig. 7 ▸ *d*). The UV–visible spectrum of this mutant was slightly red shifted to 584 nm compared with 579 nm in gfasPurple (Fig. 7 ▸ *e*), but not as far as 588 nm as in amilCP or 594 nm in the blue chromoprotein aeBlue (Tamayo-Nuñez *et al.*, 2020 ▸). A second smaller peak was also observed at 514 nm. Of these mutated residues, only Ser61 is located at the chromophore-binding site. Hence, we produced a single mutant S61I and two double mutants (S61I+P130S and S61I+G167D) to assess the contributions of each of these substitutions to colour change. As expected, the S61I mutation alone was sufficient for the colour change, producing a visibly darker pigmentation with the same colour as seen in the triple S61I+P130S+G167D mutant in *E. coli* colonies (Fig. 7 ▸ *d*).

Previous work showed that mutation of Ser61 in gfasPurple to cysteine as in amilCP causes a similar red shift in the UV spectra to 584 nm as observed in the S61I mutation, changing the visible colour towards a bluish-violet (Alieva *et al.*, 2008 ▸). However, the deep indigo of amilCP is only achieved with an additional S175T mutation (Alieva *et al.*, 2008 ▸). Free cysteine residues within proteins have a strongly hydrophobic nature (Nagano *et al.*, 1999 ▸; Iyer & Mahalakshmi, 2019 ▸) and hence could behave similarly to isoleucine in the large hydrophobic pocket surrounding position 61 (Fig. 7 ▸ *f*).

## 4. Concluding remarks

By structurally characterizing and comparing four chromoproteins, we have highlighted features in these proteins that are of interest to protein engineers and synthetic biologists to aid the incorporation of chromoproteins into various applications. Most notably, we have highlighted a conserved dimeric interface, which includes a network of water molecules linking a chromophore-interacting methionine residue in both chains of the dimer. Dimerization of these proteins seems to be important for their pigmentation, which has direct implications for their application in synthetic biology. Certainly, it limits their use as biological markers by fusing with proteins that also need to oligomerize themselves, as this can cause protein aggregation, interfere with normal protein function, prevent localization or generate false negatives due to lack of pigmentation (Olenych *et al.*, 2006 ▸). For fusion with monomeric target proteins, successful pigmentation is more likely to be achieved by linking at the N-terminus of the chromoprotein, as this is less likely to interfere with dimerization. Another promising use of chromoproteins is as FRET acceptors in fluorescent imaging applications (Murakoshi *et al.*, 2019 ▸), which is also prone to artefacts due to oligomerization and can lead to complicated data sets (Olenych *et al.*, 2006 ▸). The structures presented here may provide insight into future work in producing and engineering monomeric chromoprotein variants that are more amenable for use as biological tags and reporters.

While previously characterized chromoproteins contain *N*-acylimine formation in the peptide bond preceding the chromophore, this is a not a conserved feature in these proteins as spisPink instead contains *N*-acylamine, which is more commonly found in blue and green fluorescent proteins. This is due to its closer homology to blue and green fluorescent proteins, whereas gfasPurple, amilCP and eforRed are more homologous to the ‘dsRed-like’ red fluorescent proteins (Alieva *et al.*, 2008 ▸; Lapshin *et al.*, 2015 ▸). Despite this, the common evolutionary ancestry of all four chromoproteins is highlighted by the conserved structural features for ligand binding and dimer formation.

Finally, point mutations in the chromophore-binding site of gfasPurple to the same or similar residues as found in amilCP and spisPink create similar colour changes as the native proteins. Differently coloured mutants of the same chromoprotein tend to have similar fitness costs to host cells and are easier to use in comparative studies, although mutations at the chromophore-binding site can frequently lead to a loss of colour instead of change (Liljeruhm *et al.*, 2018 ▸). Hence, chromophore-binding site mutations inspired by other chromoproteins could be useful in generating a palette of mutants of the same protein with similar characteristics and fitness costs in cell-based applications.

## Supplementary Material

PDB reference: gfasPurple, 7swr

PDB reference: amilCP, 7sws

PDB reference: eforRED, 7swt

PDB reference: spisPINK, 7swu

Supporting information file. DOI: 10.1107/S2059798322002625/jb5043sup1.pdf

## Figures and Tables

**Table 1:** Data-collection and refinement statistics using anisotropic high-resolution cutoffs Values in parentheses are for the highest resolution shell.

	gfasPurple	amilCP	eforRED	spisPINK
PDB code	7swr	7sws	7swt	7swu
Data reduction				
Space group	*P*4_2_22	*P*12_1_1	*P*2_1_2_1_2_1_	*P*12_1_1
*a*, *b*, *c* (Å)	92.1, 92.1, 78.5	71.5, 131.7, 93.7	73.4, 75.9, 175.8	71.8, 83.7, 88.5
α, β, γ (°)	90, 90, 90	90, 100.8, 90	90, 90, 90	90, 96.8, 90
Resolution (Å)	92.1–1.39 (1.47–1.39)	92.0–1.64 (1.80–1.64)	87.9–2.01 (2.22–2.01)	87.4–1.44 (1.54–1.44)
*R*_meas_	0.125 (2.21)	0.12 (1.25)	0.19 (1.36)	0.14 (1.73)
*R*_p.i.m._	0.018 (0.414)	0.045 (0.458)	0.076 (0.522)	0.053 (0.65)
〈*I*/σ(*I*)〉	21.6 (1.7)	11.5 (1.7)	12.4 (1.9)	8.8 (1.3)
CC_1/2_	1.000 (0.708)	0.990 (0.665)	0.998 (0.718)	0.997 (0.523)
Completeness (ellipsoidal) (%)	96.1 (71.7)	87.2 (98.0)	87.5 (87.8)	94.1 (56.9)
Multiplicity	46.4 (29.2)	7.1 (7.3)	13.4 (12.8)	7.0 (7.1)
Refinement				
Resolution (Å)	19.93–1.39	92.0–1.64	87.9–2.01	87.8–1.44
No. of reflections	56516	108876	34510	157325
*R*_work_/*R*_free_	0.1664/0.1776	0.1943/0.2178	0.2090/0.2502	0.1754/0.1933
No. of atoms				
Protein	3616	31008	13999	14094
Ligand/ion	41	322	152	180
Water	394	1136	307	905
*B* factors (Å^2^)				
Protein	17.5	25.9	36.7	19.8
Ligand/ion	15.4	20.3	35.9	14.9
Water	38.2	24.9	22.4	33.9
R.m.s. deviations				
Bond lengths (Å)	0.01	0.01	0.01	0.01
Bond angles (°)	1.27	1.03	1.11	1.07
