# Supplementary material for: Over the rainbow: structural characterization of the chromoproteins gfasPurple, amilCP, spisPink and eforRed
Source: Acta Crystallogr D Struct Biol. 2022 Apr 8;78(Pt 5):599–612. doi: 10.1107/S2059798322002625 (PMC9063845; doi:10.1107/S2059798322002625)
Supplement: Supplementary file 1 [file d-78-00599-sup1.pdf]

STRUCTURAL BIOLOGY

Volume 78 (2022)

Supporting information for article:

Over the rainbow: structural characterization of the chromoproteins gfasPurple, amilCP, spisPink and eforRed

F. Hafna Ahmed, Alessandro T. Caputo, Nigel G. French, Thomas S. Peat, Jason Whitfield, Andrew C. Warden, Janet Newman and Colin Scott

Table S1 Crystallization and data collection information

	gfasPurple	amilCP	eforRED	spisPINK
**Crystallization**				
Condition	20% (w/v) PEG 3350, 0.2 M potassium thiocyanate	20% (w/v) PEG 3350, 0.1 M sodium bromide, 0.1 M bis-tris propane chloride (pH 6.5)	20% (w/v) polyethylene glycol 8000, 0.2 M sodium acetate, 0.1 M sodium MES (pH 6)	20 % (w/v) polyethylene glycol 3350, 0.2 M potassium nitrate
Temperature (K)	293	293	293	293
Cryoprotectant	15% glycerol in crystallization condition	15% glycerol in crystallization condition	15% glycerol in crystallization condition	15% glycerol in crystallization condition
**Data collection**				
Beamline	Australian Synchrotron MX2	Australian Synchrotron MX1	Australian Synchrotron MX1	Australian Synchrotron MX1
Wavelength (Å)	0.95374	0.95372	0.95372	0.95372
Temperature (K)	100	100	100	100
Detector	Dectris Eiger X 16M	Dectris Eiger X 9M	Dectris Eiger X 9M	Dectris Eiger X 16M

Table S2 PDBePISA results for interfaces a/b and a/c in figure 3

**interface**	**Molecule 1 no. of residues**	**Molecule 2 no. of residues**	**interface area (Å^2)**	**Interface $\Delta G_{solvation}$ (kcal/mol)**	**Interface $\Delta G_{solvation}$ p-value$^{\$}$**	**No. of H-bonds**	**No. of salt bridges**	**CSS***
**a/c**								
**spisPINK**	33	31	1138.5	-6.0	0.477	15	20	0.8
**eforRED**	36	38	1368	-10.0	0.384	0	14	0.1$^{\#}$
**gfasPURPLE**	25	25	1061.2	-7.9	0.365	12	8	0.2
**amilCP**	31	30	1248.4	-11.2	0.369	0	8	0.4
**a/b**								
**spisPINK**	29	29	872.7	-7.0	0.335	4	0	0.03
**eforRED**	34	35	1005.6	-2.2	0.703	5	3	0
**gfasPURPLE**	25	25	791.6	-5.8	0.371	11	0	0.1
**amilCP**	27	26	794.4	-7.0	0.321	0	0	0.2

* Complexation Significance Score (CSS), which is the maximal fraction of the total free energy of binding that belongs to the interface in stable assemblies of the protein. Higher values indicate more likelihood of significance for assembly formation.

$^{\#}$When the calculated stability is in the intermittent range of complexation criteria, the CSS is decreased by factor of 10.

$^{\$}$P-value of the observed solvation free energy gain ($\Delta G_{solvation}$), which is the probability of getting a lower than observed $\Delta G_{solvation}$ for the interface when atoms are picked randomly from the protein surface, such as to amount to the observed interface area. It measures interface specificity, or how unique it is in energy terms.

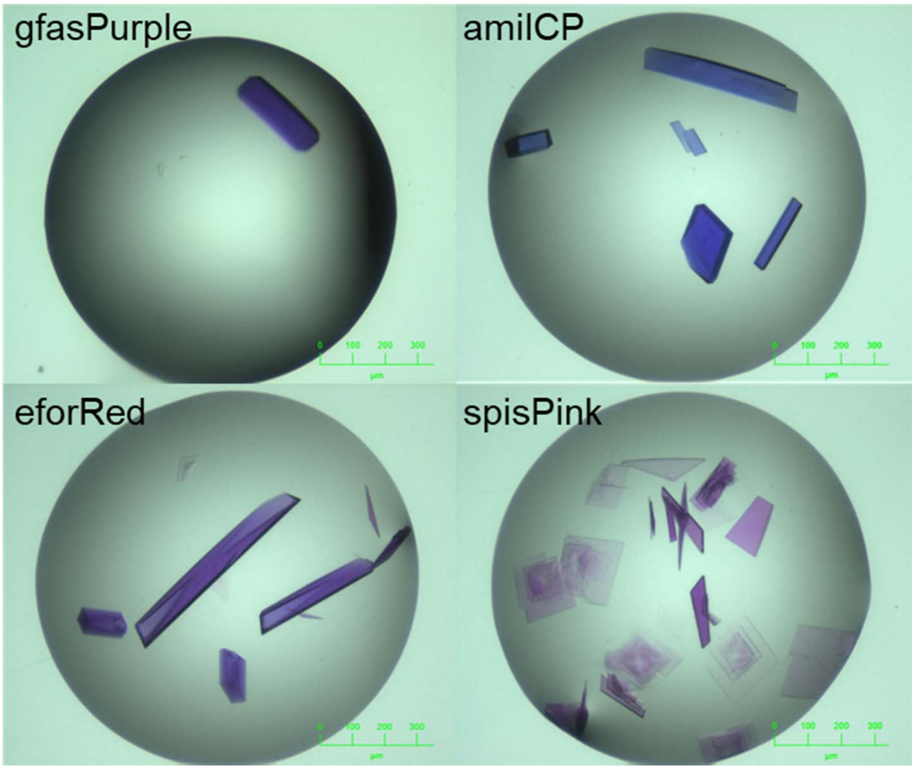

Figure S1 Crystals of the chromoproteins. Crystallisation conditions are provided in SI Table 1.

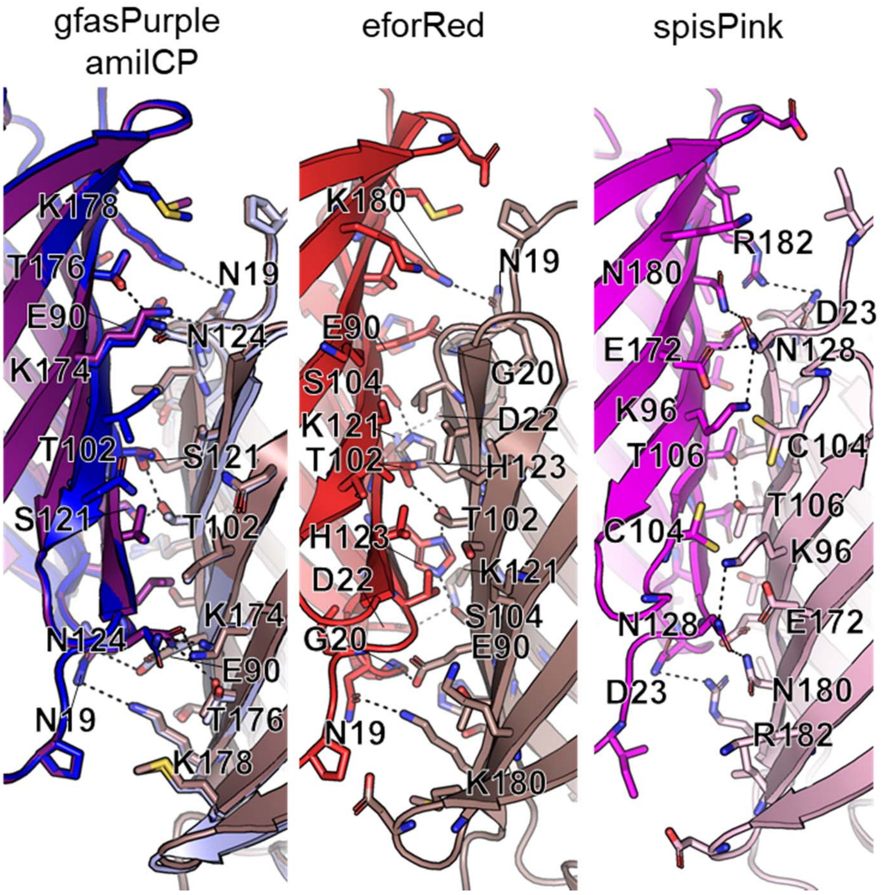

Figure S2 Interactions at the a/b interface. Two alternate conformations for C104 in the spisPink structure are shown.

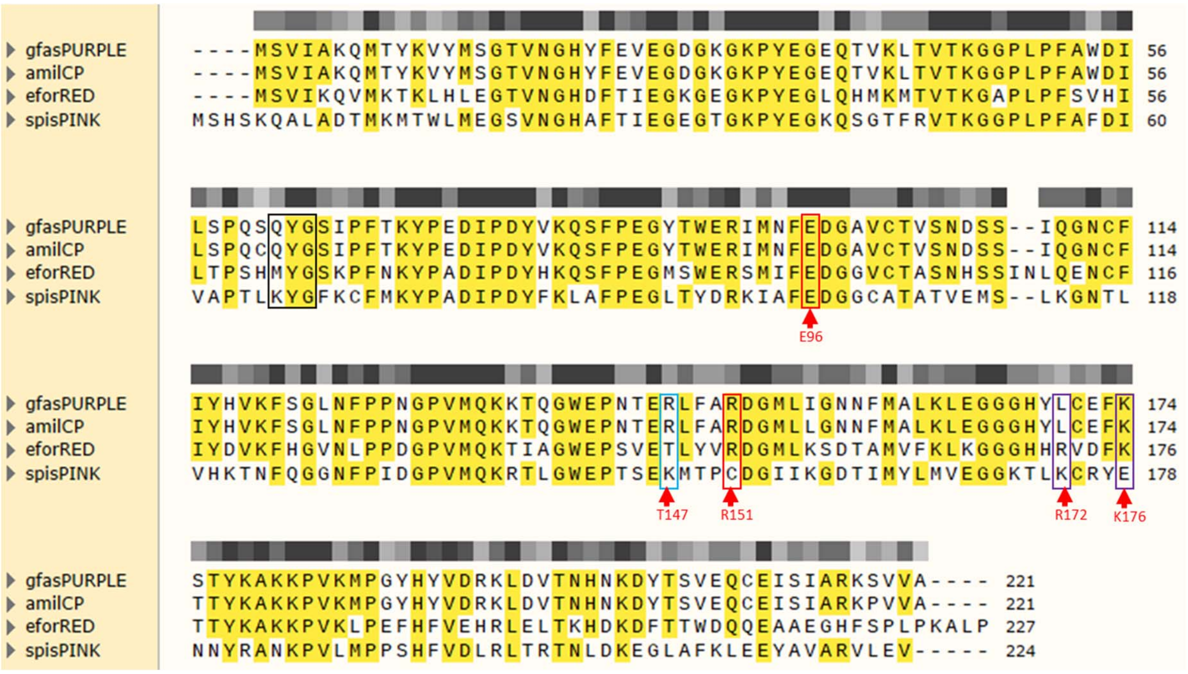

Figure S3 Sequence alignment of chromoproteins in this study. The pairs of conserved residues that form ionic interactions at the a/c dimer interface are highlighted in same colours, where the residue number references are from eforRed. Residues highlighted in blue interact with the protein backbone of the opposite chain in the dimer. The tri-peptide that forms the chromophore is highlighted in black.

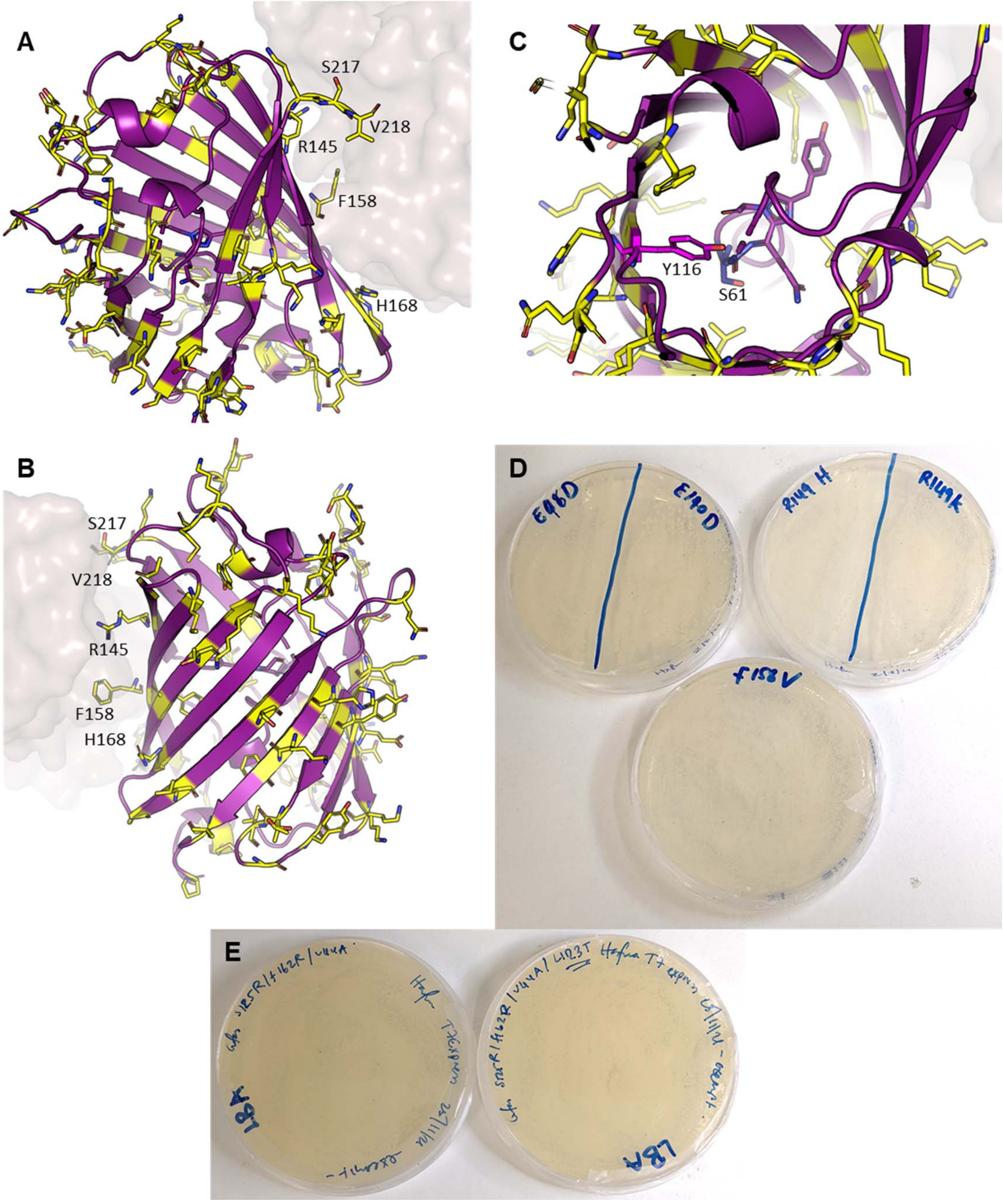

Figure S4 Mutating the a/c dimer interface of gfasPurple. **A.** Random mutation accumulation positions demonstrating reduced frequency of mutations at the a/c dimer interface. Residues that were mutated in any coloured variants are shown as sticks in yellow, and residues out of these at the a/c interface are labelled. **B.** Same as panel **A** but viewed from the opposite side. **C.** Same as panel A. but viewed into the β-barrel. Mutations in chromophore interacting residues that lead to colour changes are shown in pink or blue. **D.** Colourless *E. coli* colonies resulting from expressing gfasPurple variants with point mutations at the a/c dimer interface. Top left to right: E96D, E140D, R149H and R149K, Bottom: F158V. **E**. Colourless *E. coli* colonies resulting from replicating the monomerising mutations from Rtms5 in gfasPurple (Pettikiriarachchi *et al.*, 2012). Left: S125R+I62R+V44A, right: S125R+I62R+V44A+L123T.

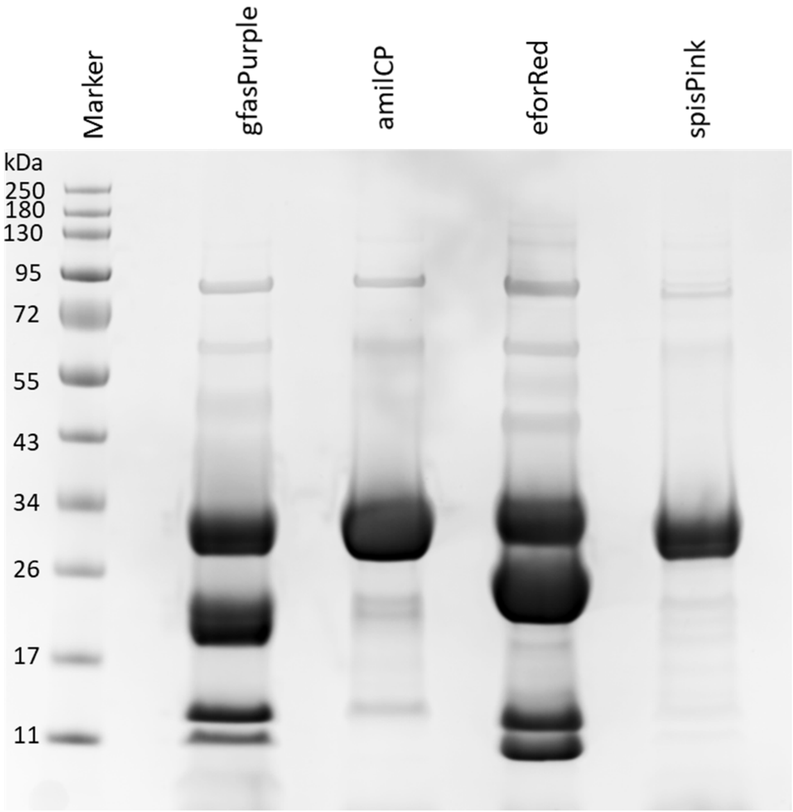

Figure S5 SDS page gel showing acylimine formation where the denatured protein is hydrolysed at the chromophore to ~18 and ~9 kDa fragments. Proteins contain a 6xhis-tag followed by a TEV protease cleavage site.

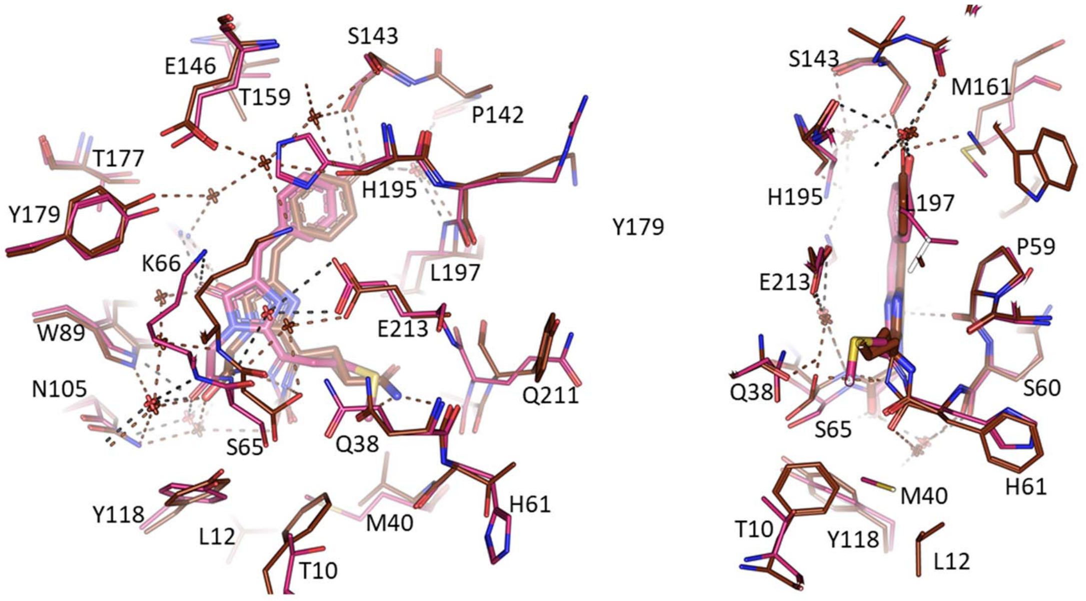

Figure S6 Comparison of the chromophore binding sites of eforRED (red) and dsRed (brown, PDB ID: 1ZGO, Wall et al., 2000), showing a front and side view. Residue labels correspond to eforRed.

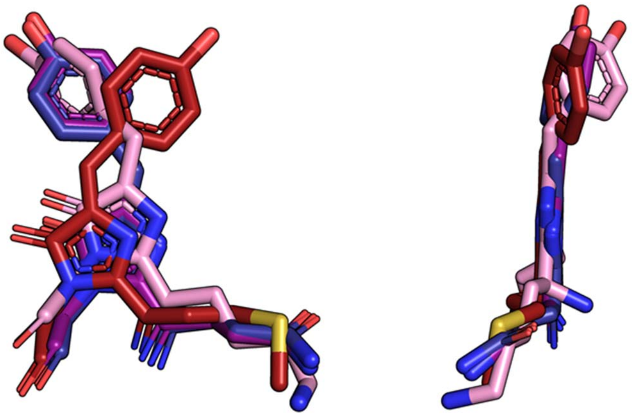

Figure S7 Overlay of the chromophores in gfasPurple, amilCP, eforRed and spisPink, showing their position in the binding site relative to each other when the protein backbones (not shown) are aligned.

References

Pettikiriarachchi, A., Gong, L., Perugini, M. A., Devenish, R. J. & Prescott, M. (2012). PLoS One. 7, e41028.

Wall, M. A., Socolich, M. & Ranganathan, R. (2000). Nat. Struct. Biol. 2000 712. 7, 1133–1138.
